# Cross-cultural adaptation and validation of Malay version of Rapid Estimate of Adult Literacy in Dentistry (MREALD-30) among Orang Asli population in Malaysia

**DOI:** 10.1186/s12903-021-01866-9

**Published:** 2021-10-12

**Authors:** Avita Rath, Melissa Wong, Claudio Mendes Pannuti, Priyadarshini Hesarghatta Ramamurthy, Bennete Fernandes, Amelia Shelton, Khairiyah Abdul Muttalib

**Affiliations:** 1 Faculty of Dentistry, SEGi University, No. 9 Jalan Teknologi PJU5, 47810 Kota Damansara, Selangor Malaysia; 2 Departamento de Estomatologia, University of São Paulo, São Paulo, Brazil; 3 Ministry of Health, Putrajaya, Malaysia

**Keywords:** REALD-30, Oral health, Health literacy, Psychometric evaluation, Malay, Malaysia, Dental, Indigenous people, Orang Asli

## Abstract

**Background:**

The aim of this study was to adapt, translate and validate the Rapid Estimate of Adult Literacy in Dentistry (MREALD-30) instrument for the Orang Asli population in Malaysia.

**Methods:**

After translation and cross-cultural adaptation, interviews were conducted with 326 participants of the Temuan tribe from village Kampung Tering in Johol, Negeri Sembilan, Malaysia. The instrument's validity was assessed using the scores of MREALD-30, which were compared based on occupation, monthly household income, educational attainment, general literacy, use of dental services, and three dental outcomes. A questionnaire containing socio-behavioral information and validated Malay Oral Health Impact Profile (M-OHIP-14) was also administered. The reliability of the MREALD-30 was assessed by re-administering it to 30 subjects after two weeks. Its correlations evaluated convergent and discriminative validity of MREALD-30 with the level of education and dental visiting habits, monthly household income, respectively. Predictive validity was assessed with M-OHIP-14, while construct validity was evaluated by exploratory factor analysis using the Rasch model.

**Results:**

The internal consistency of the MREALD-30 measured by Cronbach's alpha was 0.89. The test–retest reliability was excellent (ICC 0.95, k = 0.85). MREALD-30 exhibited good construct validity. Rasch analysis showed two factors, and infit mean-square statistics for MREALD-30 were all within the desired range of 0.50–2.0. The discriminant validity and predictive validity were statistically significant (*p* < 0.05).

**Conclusions:**

MREALD-30 showed very strong reliability, good construct, discriminant, and predictive validity, but poor convergent validity. Overall, it showed good psychometric properties and can be used in these community settings.

**Supplementary Information:**

The online version contains supplementary material available at 10.1186/s12903-021-01866-9.

## Background

Oral health is essential to the maintenance of a good quality of life. Nevertheless, particularly amongst underprivileged and neglected populations, most of the preventable oral disease remains rampant [1]. One of the causes of health disparities is the inequality of access to oral health services and consequent improper utilization of the latest health care systems [2–4]. The United Nations stated a few causes, which were insufficient state funding accompanied by high expenses, substandard quality of services in rural geographical areas, racism or bias towards indigenous populations’ culture, knowledge and environment [5]. This can be observed as the majority of the indigenous populations are situated far from the urban populations and nearby facilities [6].

Oral health literacy and oral health outcomes have been extensively studied. However, they have yet to be studied amongst disadvantaged groups such as the Orang Asli population. Functional literacy is not only related to reading and writing, but it is also established on ones’ comprehension of the etiological factors of poor oral health, persistent and motivated oral self-care behaviours, and communication with oral health care providers, constructing the foundation to a sound oral health quality of life [7, 8]. The complexity of the terms involved in communication has shown to be a setback in improving oral health, which can be overcome by focusing on oral health literacy [1, 9]. In this context, word recognition tools have demonstrated a strong association between an individuals’ established reading ability and reading comprehension skills [10]. Evidence from a previous study suggests that this person may face difficulties with comprehension when someone is struggling to pronounce the given dental-related words [11]. Hence the need to measure and improve the dental literacy skills of the community is crucial as a healthy oral health status benefits the overall health [12].

Many instruments have been used to assess dental literacy in adults. The Rapid Estimate of Adult Literacy in Dentistry (REALD-30), developed by Lee and colleagues, is the first and simplest oral health literacy (OHL) instrument [13, 14]. This instrument comprises thirty commonly used dental terminologies [13, 14]. These words are arranged in increasing order of difficulty, awarding one point for each correctly pronounced word, and the total overall score is added [12]. The instruments’ reliability and validity in measuring dental health literacy were measured among adults [14–16]. Nonetheless, the beneficial outcomes in determining the literacy levels are restricted to a particular population. Consequently, utilizing a psychometric analysis instrument provides an upper hand in evaluating populations of various cultures on a global scale [12].

In order to study the level of literacy of a specific population, it is necessary to develop an instrument in their native language that would aid the healthcare providers to understand the level of oral health knowledge and communication among the community and, in turn, facilitate implementing strategies to improvise it. So far, five studies have been conducted on oral health literacy in the Malaysian population [17–21]. The mentioned studies translated and converted OHL instruments from English to the Malay language, where the components of reading or comprehension were incorporated [17]. However, none of the studies has focused on the indigenous population of the country. The Orang Asli are the indigenous people of Peninsular Malaysia. As of 2015, an estimated 13.8% of Malaysia’s population were represented by the indigenous people residing in East and Peninsular Malaysia, segregated from the homogenous population group [22]. The Orang Asli were descendants of ancient inhabitants of the Malay Peninsula, consisting of at least nineteen different subgroups and tribes. The three major tribes are Negrito, Senoi and Proto-Malay [23]. Most of the challenges faced by the Orang Asli includes financial support, access to health care facilities, malnutrition and a lack of education dimensions [24].

To achieve the National Oral Health Goals 2020 with improved oral health status among Malaysians, it is of utmost importance to include its indigenous population [25]. Failing to do so would prove to be inadequate to address the afore-mentioned national agenda. Therefore, this study aimed to translate, perform the cross-cultural adaptation of the Rapid Estimate of Adult Literacy in Dentistry to Malay language (MREAL-30) and test the reliability and validity of this version for the Orang Asli community in Malaysia.

## Methods

### Study population

The target population (n = 326) of the present study constituted the Temuan tribe of Kampung Tering, located in Johol, Negeri Sembilan, Malaysia. Formal approval was obtained from the Ethics committee, SEGi University, Malaysia (No.SEGiIRF/2016-24/FOD-9/99). Additional clearance was also obtained from the Ministry of Health for Negeri Sembilan state, the Malaysia Department of Orang Asli Development (JAKOA), and the District Health Office of Johol. The methods involve in this study were performed in accordance with the Decleration of Helsinki [26]. The study took place as a part of a community awareness project between January 2017 till January 2019.

A free, prior, written and informed consent form was obtained from the village head of the community and all the participants before proceeding with the study. Participants with limited reading ability had consent forms and the words in MREALD-30 and MOHIP-14 read to them. The word recognition was scored as zero for these participants but word comprehension were assessed accordingly.

### Cross-cultural adaptation of MREALD-30

The original REALD-30 instrument was adapted and translated to the Orang Asli population by two independent native speakers fluent in Malay and English (A.S, K.E) [27]. The translated version was evaluated in a ‘double-blind’ approach by both the translator and a back translator, who does not know the original text.

An expert panel comprising three bilingual individuals (two were dental professionals) (J.A, M.W, K.M) was assembled to resolve any inconsistency between the translated versions. Additionally, an independent professional translator back-translated the Malay version into English to further confirmed that no discrepancies were found between the original and back-translated English versions of MREALD-30. Hence the synthesis of the MREALD-30 instrument was developed as a result of this process. Before the commencement of the main study, a pilot study was conducted with 30 subjects (aged over 18 years) who fulfilled the inclusion criteria and consent were obtained for the clarity assessment. Each participants was provided with the finalized version of MREALD-30. Feedback was obtained regarding the difficulties in understanding the items, and any required changes were made accordingly. The participants were recalled after two weeks for reliability analysis. The translated tool did not need any major adjustment based on the pilot study and it was used as it is for the main study.

### Instruments used

The MREALD-30 instrument (Additional file 1) was used to collect data during face-to-face interviews to evaluate oral health literacy. The participants were firstly asked to read aloud each MREALD-30 word (word recognition) and then explain its meaning (comprehension). The MREALD-30 was scored by assigning one point for each word correctly pronounced (MREALD-WR) and one point was awarded to the participants by the interviewers for explaining each word close to the original definition (MREALD-COMP). The total scores for each section ranged from 0 (lowest) to 30 points (highest level of oral health literacy) [14].

The oral health-related quality of life (OHRQoL) was assessed using the oral health impact profile OHIP-14, an instrument based on the perception of problems with the teeth and mouth in the last month [12, 27, 28]. It is the most frequently used instrument in evaluating the correlation between oral health status and quality of life by measuring the impact of oral condition: functional limitation, physical pain and disability, psychological discomfort and disability, social disability, and handicap [12]. The answers were marked on a five-point Likert scale as follows: 'very often' = 4, 'fairly often' = 3, 'occasionally' = 2, 'hardly ever' = 1, and 'never/I don't know' = 0. The scores were added with the total scores ranging from 0 to 56. Participants with a lower score on the OHIP-14 indicate a higher OHRQoL.

Lastly, the demographic characteristics (age, gender, years of education, and dental visiting habits) and participants’ self-rated oral health status (recorded on a five-point Likert scale: excellent, very good, good, fair, and poor) were recorded [14, 27]. Questions regarding the causes of dental caries and gum disease were used to assess the oral health knowledge of each participant.

### Statistical analyses

Descriptive analyses were performed (mean, median, standard deviation, and total MREALD-30, M-OHIP-14 scores) to describe the main background characteristics of the participants. Independent t-test was carried out to compare the genders regarding the income, education level and frequency of dental visit.

### Reliability

Two types of reliability were assessed, which were: test–retest and internal consistency. The test–retest (or intra-rater) performance was evaluated by making 30 subjects read the words twice. The interval between the scoring sessions was two weeks. Test–retest reliability was calculated with intra-class correlation coefficient (ICC); (ICC agreements; < 0.40-poor to fair, 0.41–0.60-moderate, 0.61–0.80-good, > 0.80-excellent), using a mixed-effects model and Kappa coefficient, in order to assess agreement on a word-by-word basis; (Kappa; < 0.20-poor; 0.21–0.40-fair; 0.41–0.60-moderate; 0.61–0.80-substantial; 0.81–1.00-almost perfect) [29–31]. Cronbach’s alpha was used to measure the internal consistency of the items in the MREALD-30 total score.

### Validity

Principal Component Analysis (PCA) with varimax rotation was performed to evaluate the construct validity of MREALD-30 to ensure that it follows the one-factor solution. The Kaiser–Meyer–Olkin (KMO) was used to measure the adequacy of the data for factor analysis along with Barlett’s test of sphericity. The decision to retain the factors were based on Kaiser’s criterion with eigenvalues of > 1, the characteristics of the screen plot of eigenvalues, at least 3 items substantially loading (> 0.50) on a factor and meaningful interpretability. These data were analyzed using IBM Statistical Package for the Social Sciences (SPSS) version 26 for Windows (SPSS Inc. Chicago. IL, USA).

Convergent validity was assessed by correlating the MREALD-30 scores with the level of educational attainment, while discriminant validity was tested by comparing MREALD-30 scores according to dental visits and monthly household income.

In terms of predictive validity, the MREALD-30 scores were correlated to the impact of oral conditions on quality of life using M-OHIP-14. The influence of MREALD-30 on these oral health-related variables could be explained by the fact that, according to the model developed by Guo et al., higher health literacy levels were associated with better patient-dentist communication, which in turn corresponded with being a regular (rather than problem-oriented) dental care seeker, and finally being these better dental care pattern associated with better self-rated oral health [32].

As MREALD-30 scores were not normally distributed, the nonparametric Wilcoxon test, Mann–Whitney test, and Spearman's correlation coefficient were used in these analyses. The chi-square, Kruskal–Wallis and Mann–Whitney tests were used to evaluate the differences in the total sum REALD scores between different sub-groups. IBM SPSS Statistics, version 26, Armonk, NY: IBM Corp. software were used to perform the descriptive analyses. A significance level of 5% was used for all tests.

## Results

The study participants comprised 326 adults with a mean age of 38.1 $\pm$ 13 years with Malay as their first language. An encouraging 100% response rate was observed for this survey. Table 1 shows the distributions of participants’ basic characteristics. Of the 326 participants, 99 (30.4%) were males, and 227 (69.6%) were females. More than half (55.8%) of the subjects had completed their secondary education at the education level. No significant difference between the genders in terms of education, income, and dental visits was observed. (Table 2).

**Table 1:** Background characteristics of the study population (n = 326)

Characteristics	N(%)	Mean(SD)
Gender		
Male	99(30.4)	
Female	227(69.6)	
Age		38.1 (13)
Educational status		
No formal training	62(19)	
Completed primary school	48(14.7)	
Completed secondary school	184(56.4)	
Completed college or university	32(9.8)	
Income		
No income	15(4.6)	
500–1000 MYR	152(46.6)	
1500–2000 MYR	132(40.5)	
More than 2000 MYR	27(8.3)	
Dental visits		
< than 6 months	70(21.5)	
6–12 months	21(6.4)	
1–2 years	80(24.5)	
2–5 years	38(11.7)	
> 5 years	82(25.2)	
Never been to a dentist	35(10.7)	

N = number of participants

**Table 2:** Comparison of genders to income, education level and frequency of dental visit using independent t-test (equality of means assumed)

	Male	Female	t-test *p-*value
Monthly Income in Malaysian Ringgits, RM	1.48 (0.6)	1.54(0.7)	0.508
Education level	3.46(1.2)	3.48(1.4)	0.907
Number of Dental visits	3.54(1.5)	3.4(1.7)	0.489

**Fig. 1:**
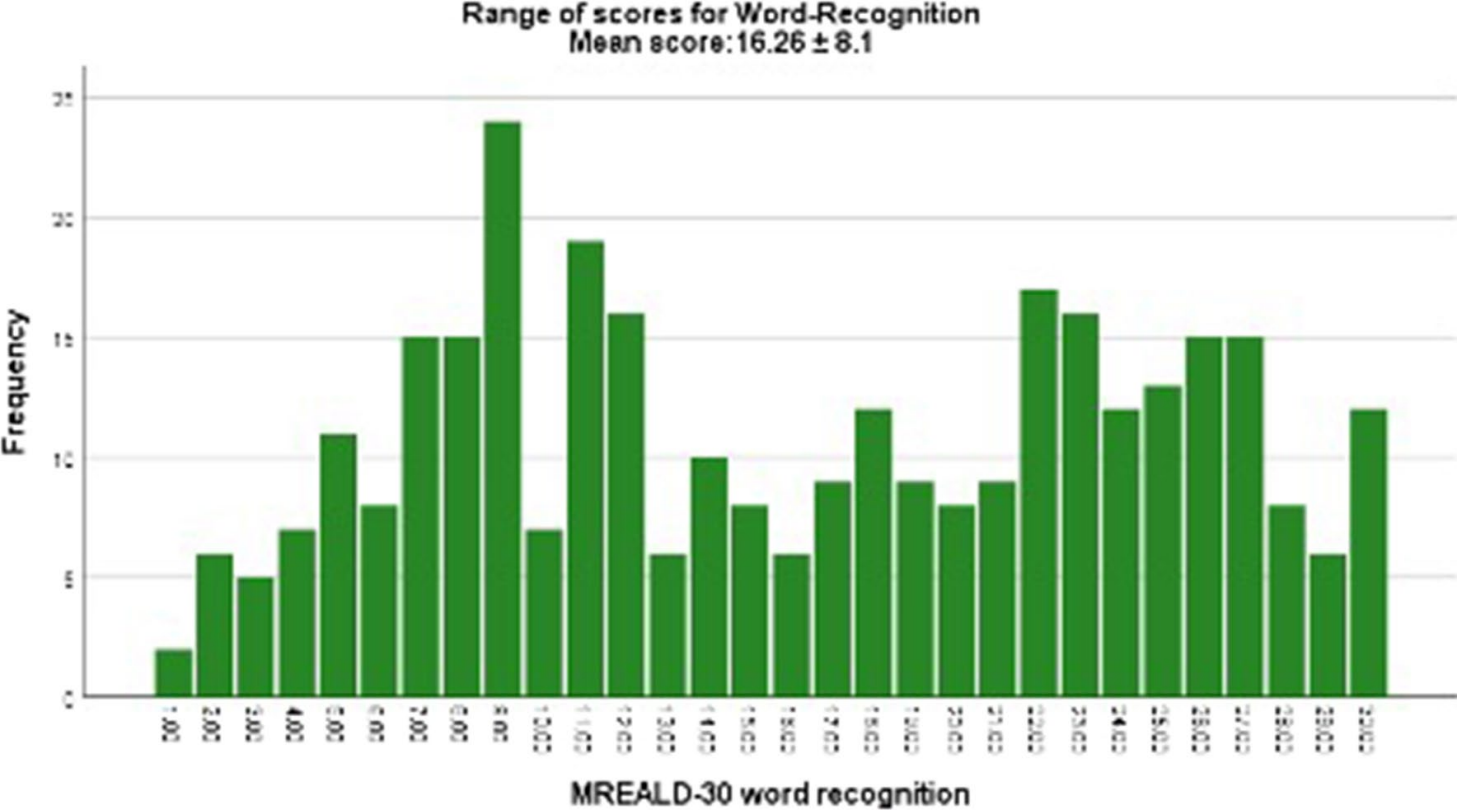
Range of scores for MREALD-30 Word Recognition (MREALD-WR)

**Fig. 2:**
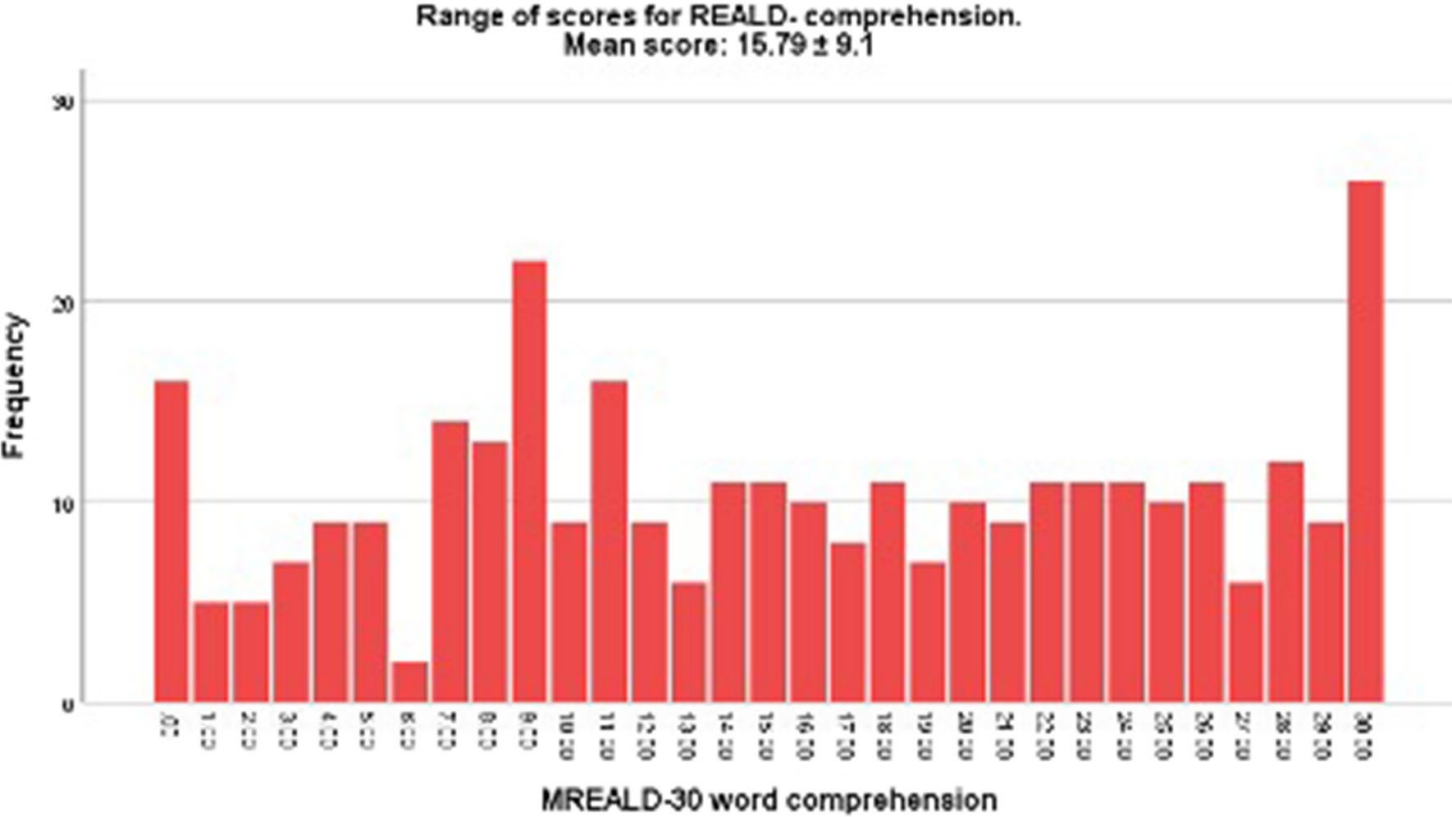
Range of scores for MREALD-30 Comprehension (MREALD-COMP)

The mean total MREALD-30 score was 32 (SD: 15.5), and the mean OHIP-14 score was 17.9(SD: 2.3). Figure 1 shows the distribution of the MREALD-Word Recognition (MREALD-WR) scores which ranged from 1–30 with a mean of 16.26 $\pm$ 8.1. Figure 2 presents the distribution of the MREALD-Comprehension (MREALD-COMP) with a range of 0–30 and mean of 15.79 $\pm$ 9.1. The results demonstrated that the participants scored approximately the same for both components, with word recognition slightly higher. MREALD-30 demonstrated good internal reliability. Cronbach's alpha was 0.89 that ranged to 0.92 when words were deleted individually. The analysis of test–retest reliability demonstrated very strong reproducibility [ICC = 0.95 (95% CI: 0.94 to 0.96)] and almost perfect Kappa coefficient (k = 0.85).

Discriminant validity was determined by comparing MREALD-30 scores according to the history of dental visits (Mann Whitney, *p* = 0.03) and monthly household income (r_s_ = 0.32; *p* < 0.001), with statistically significant differences in the scores among the different groups. The most frequent reason concerning these dental visits, especially for the male participants, was when they experienced pain alone. They also revealed had higher M-OHIP-14 scores than women (*p* < 0.05). Further, women demonstrated significantly greater MREALD-30 scores than males (Mann Whitney test, *p* < 0.001).

However, in convergent validity, MREALD-30 was very weak, as educational attainment levels were nonsignificant (r_s_ = 0.05; *p* = 0.3). As far as predictive validity was concerned, a significant but weak negative correlation was found between the MREALD-30 and OHIP-14 scores (r_s_ = − 0.13; *p* = 0.014). Monthly household income was significantly correlated with MREALD-30 scores (r_s_ = 0.32; *p* < 0.01).

Construct validity of MREALD-30 was determined by PCA with varimax rotation, using the Kaiser–Meyer–Olkin (KMO) measure of sampling adequacy and Barett’s test of sphericity. The KMO value was 0.841 and the Barlett’s test for sphericity showed significance (*p* < 0.001). The analysis carried out indicated MREAL-30 was suitable for PCA. The PCA revealed 9 factors with eigenvalues > 1, making up 64.18% of the total variance with majority of the items containing fewer than 3 items. Furthermore, 10 items were extracted from the instrument as the loading factors were low (< 0.50). The 1st factor accounted for 27.68% of the total variance (items 4,9,12,15,16,17,18,20,21,24,25,26,30; Alpha = 0.82; eigenvalue = 8.28) was regarded as dental terms used by patients on a daily basis. The 2nd factor that comprises 7.44% of the total variance was reported as technical dental terms used by dental health professionals in their daily practice (items 1,2,22,23,27,28,29; Alpha = 0.73; eigenvalue = 2.23). Based on the criterion, the ratio greater than 4.0 indicates a unidimensional instrument. The ratio of the 1st and 2nd eigenvalue was (8.28/2.23 = 3.71) showed that MREALD-30 was multidimensional [33].

Using Rasch analysis, it was seen that the infit mean-square (MNSQ) statistics remained within the acceptable range of 0.50–2.0. Since the outfit mean-square (MNSQ) statistics are more sensitive to outliers, these items were outside the range (restoration, denture, sugar, gingiva, smoking, halitosis, caries, floss, brush and extraction). The reliability estimates were 0.86, meeting the desired amounts. The person separation index met the desired 3.0 ( 2.4 to 2.9 with and without extremes, respectively). The amount of variance was 50.7%. To summarize, all items correlated positively with the estimated measure with a mean of 0.55 (0.2), with a range of 0.25 to 0.66. (Table 3).

**Table 3:** Rasch analysis of MREALD-30

	Item mean	Outfit MNSQ	Outfit ZSTD	Infit MNSQ	Infit ZSTD
Temporomandibular	0.32	1.32	0.62	1.10	0.52
Hypoplasia	0.34	0.51	− 0.15	0.76	− 1.38
Plaque	0.40	1.35	0.61	0.85	− 1.04
Braces	0.47	1.54	0.80	1.39	2.30
Cellulitis	0.45	0.80	0.05	0.86	− 1.05
Apicoectomy	0.50	1.14	0.42	1.20	1.03
Fluoride	0.63	0.56	− 1.10	0.72	− 1.62
Bruxism	0.66	0.77	− 0.25	0.70	− 1.82
Pulp	0.63	1.33	0.75	0.84	− 0.90
Periodontal	0.61	0.60	− 0.80	0.86	− 0.71
Enamel	0.60	0.71	− 0.52	0.94	− 0.33
Restoration	0.88	0.52	− 0.23	0.69	− 1.13
Fistula	0.62	1.41	0.90	1.46	2.00
Sealant	0.72	1.20	0.53	1.20	1.19
Genetics	0.82	1.24	0.61	1.10	0.51
Incipient	0.81	0.51	− 0.89	0.74	− 1.08
Dentition	0.80	0.55	− 0.87	0.95	0.03
Abscess	0.82	1.22	0.52	1.11	0.44
Malocclusion	0.81	0.62	− 0.28	1.13	0.51
Denture	0.87	0.35	− 0.60	0.70	− 1.20
Gingiva	0.90	7.77	3.42	1.26	1.01
Hyperaemia	0.70	0.45	− 1.31	0.66	− 2.10
Analgesia	0.93	0.24	0.13	0.94	0.01
Sugar	0.93	0.21	− 0.38	0.67	− 0.90
Smoking	0.94	0.32	0.02	0.91	0.01
Floss	0.93	5.20	1.82	1.15	0.40
Extraction	0.92	7.58	2.03	1.20	0.60
Halitosis	0.98	0.21	0.12	0.91	0.01
Caries	0.95	0.25	0.51	0.71	− 0.40
Brush	0.99	9.05	2.73	1.13	0.42

## Discussion

The need to determine the oral health literacy (OHL) level in a population is of utmost importance. It allows dental health professionals to modify their approach and communication strategies for every patient. Oral health literacy may be a determinant of oral health [34]. Hence it is important to identify individuals with low OHL as participants in the present study were less aware and less motivated towards dental diseases. To achieve acceptable oral health status, an individual must first understand oral health in general, followed by ways to access information regarding oral health and finally recognize it. Patients with low oral health literacy will find this particular task very strenuous to accomplish. Language and culture play a significant impact on the reliability and validity of OHL instruments [35–38]. Thus, MREALD-30, a word recognition instrument, was developed from the original English version and adapted into the Malay language to assess the Orang Asli population's oral health literacy level. Evidence has provided for its validity and reliability. Cronbach's alpha of MREALD-30 was 0.89, which shows good internal consistency and it is in accordance to the original REALD-30 (α = 0.87), besides a study in Romania (α = 0.88), Saudi Arabia (α = 0.89), Brazil (α = 0.88), Thailand (α = 0.95), Chile (α = 0.87), Hong Kong (α = 0.84) and Turkey (α = 0.91) [12, 14, 15, 26, 40–43]. The test–retest reliability was assessed using ICC. The results demonstrated very strong reproducibility of the instrument (ICC = 0.95; Kappa coefficient = 0.85), in line with results from previous studies done in Romania (0.95), Saudi Arabia (0.99), Brazil (0.98), Thailand (0.99), Hong Kong (0.78) and Turkey (0.99) [12, 15, 27, 38, 39, 41].

OHIP-14 is a good instrument for testing the predictive validity of the OHL score as it provides a more comprehensive measure than self-perceived oral health status [41]. The predictive validity of MREALD-30 showed a highly significant inverse correlation with OHIP-14 in the present study. Participants who scored better in MREALD-30 had a lower OHIP-14 score, which indicates a better quality of life. These findings were similar to the results in Thai, Spain, Saudi Arabia, India, the original version, and Jamieson et al. on Indigenous Australians and American Indians. All this evidence revealed a negative correlation with OHIP-14 was statistically significant [14, 39, 43–46]. The present study demonstrated that low oral health literacy measured by MREALD-30 was correlated to poor oral health outcomes, such as change of dental health status and oral health-related quality of life (OHRQoL) measured by OHIP-14 [42]. As compared with previous study that used the similar instrument tool, the participants in the present study had a relatively lower score (mean 16.26 $\pm$ 8.1) on word recognition, in contrast to the study done on patients in a dental clinic in Connecticut, USA (mean 22.98 $\pm$ 5.1) [47]. However, the comprehension scores were slightly similar (mean 16.05 $\pm$ 4.3) [47]. Several authors from different studies have highlighted the limitation of the word recognition tool in assessing the participants OHL as it does not indicate that the person understood the meaning of the word [47–49]. Therefore, the findings from the current study supports this assertion, where the scores for MREALD-WR were higher than MREALD-COMP, indicating that the participants were able to read rather than defining the words. Hence, comprehension of the words in MREALD-30 should not be taken lightly and it should be considered in addition to word recognition.

Previous studies have observed that limited health-related knowledge is a risk indicator for poor self-reported general health. Poor general self-care behaviours are a risk indicator for poor general health-related quality of life [50, 51]. It has been reported in multiple studies that different dental service utilization patterns like a patient’s last visit to a dentist for any problem have also been linked with poor OHL and poor OHRQoL [13, 28, 38, 52, 53]. In their study, Jamieson et al. stated that subjects with lower OHL have less frequent dental visits, inferior self-rated oral health status, and more perceived treatment needs [46]. This finding contrasts with the current study, where our participants’ MREALD-30 and OHIP-14 scores were not associated with their last visit with a dentist. This is in line with the Arabic version of REALD-30 in Saudi Arabia, the first version of REALD-30. A recent systematic review observed no significant association between OHL and OHRQoL with the frequency of dental visits [12, 14, 54]. The probable explanation for the lack of association reported in the systematic review could be due to bias due to the study’s cross-sectional design. This kind of study presents limitations in the observation of the causal relationship between outcome and exposure since both are examined simultaneously [54].

The MREALD-30 scores for female participants were significantly better than the male participants in our study, which agrees with the findings reported in Romania, Persia, and Iran [38, 55, 56]. The study explains this finding with females having greater exposure to audio-visual media, one of the most common sources for distributing oral health-related information to the public [56]. When comparing the OHRQoL and the gender of participants, results demonstrated no significant correlation; however, Romanian males perceived the quality of their oral health negatively than women [38]. Regarding the convergent validity by correlating the MREALD-30 scores with the participants' educational status, no significant correlation was reported in the present study. This finding is supported by a recent study from Iran, which observed that OHL scores were independent of patients' education [34]. However, the OHL level in Romanian and Greek participants differed significantly from the participants' educational level [38, 57]. Although some literature suggests that health literacy plays an important role in those with lower education than those with higher education, the association was only partly per previous studies on OHL. Some studies state that OHL is not all about the educational level [14, 58–63]. Evidence has shown that OHL is positively correlated to information seeking, which is associated with education levels [62]. Nevertheless, health knowledge improves with health-related education programs, that can be tailored to populations with low health literacy. The Ministry of Health of Malaysia has consistently supported such kind of outreached program among different communities, especially the indigenous population, by working hand in hand with JAKOA [64]. A possible explanation for the lack of association with the education levels and MREALD score could be due to the constant exposure to oral health awareness programs by the government. However, participants' educational level should be evaluated with caution to oral health literacy. It does not accurately consider an individuals' ability to understand and comprehend a piece of written information [14]. MREALD-30 could not discriminate against participants’ OHL level and socioeconomic status as they were not significantly correlated, which conforms with the study done in Persia and Iran [34, 55]. However, a study conducted in Brazil confirmed that Brazilians with a higher household income had a significantly higher degree in oral health literacy as they were able to seek dental treatments. Other studies reported that subjects in the low-income group with a low degree of dental health literacy did not often visit the dental facilities [27, 52, 53].

Regarding construct validity, although the Principal Component Analysis (PCA) demonstrated that MREALD-30 was multidimensional as the ratio between the first and second eigenvalue was 3.71. These findings were similar to the original study (REALD-30), BREALD-30 and TREALD-30, where there is a predominance of one factor over the other factor, with the eigenvalue of almost fourfold greater along with the presence of at least one more factor [14, 27, 41]. The authors of the REALD-30 suggest some possible explanations for the multidimensionality of dental health literacy that might be due to the differences in the reading ability and the difficulty of the words tested [14]. This explanation seems consistent when observing the words included in factor I (braces, pulp, restoration, genetics, incipient, dentition, abscess denture, gingiva sugar, smoking, floss, brush), falling under the easiest group. A hypothesis can be drawn where the presence of domains related to different difficulty levels have the ability to increase the discriminating power of a test by assessesing ones’ ability to recognize words. Nevertheless, further studies are needed to confirm and understand this hypothesis.

In context to the conceptual and semantic equivalents of MREALD-30, discussion with our advising anthropologist and the feedback of the pilot study was a crucial step in validating the translated tool. Words like *halitosis and floss* were directly translated into ‘smelly mouth’ and ‘teeth string’ for better understanding as it is used on a daily basis among Orang Asli population. However, words that have been used in English by dental practitioners, such as *fistula, enamel, periodontal, cellulitis* etc. were translated by changing their spelling according to the Malay language due to the non-existence of the specific terminology. The translation and the back translation team were successful in formulating the final translated version, which demonstrated a high clarity among the population.

It is understandable from previous studies that the two domains underlying the OHL may be related to differences in reading ability and the difficulty of the words [12, 14, 27, 65, 66]. Rasch analysis determined the individuals' ability and the difficulty of filling the questionnaire independently along the common measurements. It does not segregate our participants with the top scores [67]. Furthermore, the Rasch analysis supports the use of all items in MREALD-30 as they play a part in the measure by quantifying different literacy attributes, as denoted by appropriate mean-square estimates [68–70]. The outfit mean-square statistics are more sensitive to outliers; ten out of thirty items outside the range (restoration, denture, gingiva, sugar, smoking, floss, extraction, halitosis, caries, brush) in Rasch analysis. Misfit of items indicates a lack of association between the items in the scale, reducing the instruments’ quality. Words like ‘denture,’ ‘gingiva,’ ‘sugar,’ ‘smoking,’ ‘brush’ are commonly used terms daily, which were similar to the findings reported among the Romanian (RREALD-30), Brazilian (BREALD-30), Turkish (TREALD-30), Persian (IREALD-99) and Saudi Arabic (AREALD-30) population [12, 27, 38, 41, 55]. On the other hand, difficult or uncommon words such as ‘hyperemia,’ ‘analgesia,’ ‘extraction,’ ‘halitosis,’ ‘caries’ were challenging among the Orang Asli population in the study probably due to the lack of association with the general populations’ everyday lives and are terms commonly used by the oral health care professionals [38]. To improve the instrument's validity, misfitting items need to be discarded until no further improvement in the fit requirements was found; however, more studies need to be conducted on a larger scale [12, 65, 71]. Regarding our study, these items were not removed because the Infit mean-square statistics were acceptable, and it is more useful in our analysis [12]. Two hundred and ninety-seven participants achieved a maximum score (91.1%), and fifteen participants received a minimum score (4.6%). The Rasch model reported 50.9% of variance from our results, indicating MREALD-30 as an acceptable multidimensional instrument in line with RREALD-30, AREALD-30, IREALD-99, and BREALD-30 [12, 27, 38, 55]. Finally, all items in MREALD-30 correlated positively with the estimated measure by exhibiting a good model fit which supports the use of MREALD-30 in assessing oral health literacy on an individual and community level by identifying participants with low degrees of dental health literacy, allowing dental health administrators to develop a more appropriate educational approach [27]. Similar to findings from the previous study, our results demonstrated MREALD-30 with good psychometric properties being a rapid, simple, and reliable measure of dental health literacy among the Orang Asli population who speak Malay [27].

Nevertheless, the present study had some limitations that need to be considered. Firstly, the study was carried out among the Temuan tribe limiting the generalizability of the results to the other tribes of Orang Asli. Further studies on other tribes of the community are recommended to assess the generalizability of MREALD-30. The instruments’ sensitivity towards different community and culture would be riveting to study. Furthermore, since this was a cross-sectional study, the cause-effect relationship or even changes in participants' OHL were not assessed. Although many studies considered the instrument unrealistic with its proven validity and reliability, it is a word recognition tool. It does not appraise the function and comprehension [12, 15, 27, 34, 42, 46, 55]. Hence, several recent studies and a systemic review have stressed the importance of formulating a valid and reliable tool to evaluate the functional and conceptual literacy in oral health accurately among individuals with different levels of oral health literacy in a clinical or a community setting [27, 32, 47, 72, 73]. Our findings suggest that more investigations need to be executed in the future on different Orang Asli tribes in Malaysia to fully understand the oral health literacy across the board. Moreover, the role of oral health literacy-related outcomes on numerous sectors of poor self-related oral health needs to be addressed. This is particularly relevant in this research carried out among the Orang Asli population group from the Temuan tribe. They were groups who experienced unsatisfactory levels of both dental disease and poor oral health-related quality of life. They are at a disadvantage since they cannot always access the care they require [8]. As we can confirm from our findings, participants with a higher score on dental health literacy could better oral health status and OHRQoL [36]. Meanwhile, improving the Orang Asli population's socioeconomic conditions is generally beyond the scope of dental public health; however, improving their access to dental care by increasing the number of dental health care facilities in rural areas is achievable [74]. Identifying various individual levels and the causative factors to poorer self-rated oral health is vital in commencing the best practice and effective dental health services and programs targeting specifically the Orang Asli populations in Malaysia [74].

## Conclusions

To summarize, the cross-cultural adaption of MREALD-30 showed excellent reliability on repeated administrations and internal consistency as a oral health literacy tool among orang asli community who communicates in the Malay language. Although MREALD-30 exhibited good construct and descriptive validity, its predictive validity was poor. Our data revealed that oral health literacy can be improved with the presence of proper oral health awareness programs despite of individual education levels. The evaluation of oral health literacy was enhanced by measuring the participants’ ability in word recognition and comprehension as some may be able to recognize the words without understanding them. Finally, the Rasch model supported the use of this adaptation as each item demonstrated to have a good fit for the data. Malaysia being a diverse nation comprising of different races, future studies can be conducted among the other ethnics to assess the generalizability of MREALD-30. This tool may prove useful to analyze the oral health literacy among the Malaysian population nationwide.

## Supplementary Information

**Additional file 1**. MREALD-30 questionnaire.

## Data Availability

The datasets generated and analyzed during the current study are not publicaly available due to restriction imposed by the Ministry of Health, Malaysia and Malaysia Department of Orang Asli Development (JAKOA) but are available from the corresponding author on reasonable request. Confidentiality of the individual participants is masked as requested by Ethics.

## Abbreviations

REALD-30: Rapid Estimate of Adult Literacy in Dentistry
OHL: Oral health literacy
MREAL-30: Rapid Estimate of Adult Literacy in Dentistry to Malay language
JAKOA: Malaysia Department of Orang Asli Development
OHRQoL: Oral health-related quality of life
OHIP-14: Oral health impact profile
ICC: Intra-class correlation coefficient
PCA: Principal component analysis
KMO: Kaiser–Meyer–Olkin
SPSS: Statistical Package for the Social Sciences
MNSQ: Infit mean-square
MNSQ: Outfit mean-square
RREALD-30: Romanian Rapid Estimate of Adult Literacy in Dentistry
BREALD-30: Brazilian Rapid Estimate of Adult Literacy in Dentistry
TREALD-30: Turkish Rapid Estimate of Adult Literacy in Dentistry
IREALD-99: Persian Rapid Estimate of Adult Literacy in Dentistry
AREALD-30: Saudi Arabic Rapid Estimate of Adult Literacy in Dentistry
