# Supplementary material for: Cross-cultural adaptation and validation of Malay version of Rapid Estimate of Adult Literacy in Dentistry (MREALD-30) among Orang Asli population in Malaysia
Source: BMC Oral Health. 2021 Oct 12;21:519. doi: 10.1186/s12903-021-01866-9 (PMC8513292; doi:10.1186/s12903-021-01866-9)
Supplement: Supplementary file 1 — Additional file 1. MREALD-30 questionnaire. [file 12903_2021_1866_MOESM1_ESM.docx]

**Cross-cultural adaptation and validation of Malay version of Rapid Estimate of Adult Literacy in Dentistry (MREALD-30) among Orang Asli population in Malaysia**

**Authors:** Avita Rath, Melissa Wong Li Zheng, Claudio Pannuti, Priyadarshini HR, Bennete Fernandes, Amelia Shelton, Khairiyah Abdul Muttalib

Nama/*Name*:………………………………………………..

**(Tanda (√) di sebelah perkataan yang anda tahu dan boleh baca mengikut urutan yang diberikan)**

*Tick (√) next to the words you know and can read in the order given*.

| Words  Perkataan | I know  Saya tahu | I can read  Saya tahu baca |
| --- | --- | --- |
| Temporomandibular- Temporomandibular |  |  |
| Hypoplasia-Hipoplasia |  |  |
| Plaque-Plak |  |  |
| Braces-Pendakap gigi |  |  |
| Cellulitis-Cellulitis |  |  |
| Apicoectomy- Apikoektomi |  |  |
| Fluoride-Florida |  |  |
| Bruxism-Tabiat |  |  |
| Pulp-Pulpa |  |  |
| Periodontal-Periodontal |  |  |
| Enamel-Enamel |  |  |
| Restoration-Tampalan |  |  |
| Fistula-Fistula |  |  |
| Sealant- Sealan |  |  |
| Genetics-Genetik |  |  |
| Incipient-Permulaan |  |  |
| Dentition-Gigi |  |  |
| Abscess-nanah |  |  |
| Malocclusion-Maloklusi |  |  |
| Denture-Dentur |  |  |
| Gingiva-Gusi |  |  |
| Hyperaemia- Hiperemia |  |  |
| Analgesia-Analgesik |  |  |
| Sugar – Gula |  |  |
| Smoking- Merokok |  |  |
| Floss-Benang gigi |  |  |
| Extraction-Cabutan |  |  |
| Halitosis-Mulut berbau |  |  |
| Caries- Karies |  |  |
| Brush-Berus |  |  |

| **Total no. of words known=** |
| --- |
| **Total no. of words read=** |
| **Total=** |

**THANK YOU FOR DOING THIS SURVEY**

**TERIMA KASIH KERANA MEMBUAT KAJIAN INI**
